# Characterization of Nef expression in different brain regions of SIV-infected macaques

**DOI:** 10.1371/journal.pone.0241667

**Published:** 2020-11-02

**Authors:** Shadan S. Yarandi, Jake A. Robinson, Sarah Vakili, Martina Donadoni, Tricia H. Burdo, Ilker K. Sariyer

**Affiliations:** Department of Neuroscience, Center for Neurovirology, Lewis Katz School of Medicine at Temple University, Philadelphia, PA, United States of America; University of Texas Medical Branch at Galveston, UNITED STATES

## Abstract

**Objective:**

HIV-associated CNS dysfunction is a significant problem among people with HIV (PWH), who now live longer due to viral suppression from combined anti-retroviral therapy (ART). Over the course of infection, HIV generates toxic viral proteins and induces inflammatory cytokines that have toxic effects on neurons in the CNS. Among these viral proteins, HIV Nef has been found in neurons of postmortem brain specimens from PWH. However, the source of Nef and its impact on neuronal cell homeostasis are still elusive.

**Methods and results:**

Here, in using a simian immunodeficiency virus (SIV) infected rhesus macaque model of neuroHIV, we find SIV Nef reactivity in the frontal cortex, hippocampus and cerebellum of SIV-infected animals using immunohistochemistry (IHC). Interestingly, SIV-infected macaques treated with ART also showed frequent Nef positive cells in the cerebellum and hippocampus. Using dual quantitative RNAscope and IHC, we observed cells that were positive for Nef, but were not for SIV RNA, suggesting that Nef protein is present in cells that are not actively infected with SIV. Using cell specific markers, we observed Nef protein in microglia/macrophages and astrocytes. Importantly, we also identified a number of NeuN-positive neurons, which are not permissive to SIV infection, but contained Nef protein. Further characterization of Nef-positive neurons showed caspase 3 activation, indicating late stage apoptosis in the CNS neurons.

**Conclusions:**

Our results suggest that regardless of ART status, Nef is expressed in the brain of SIV infected macaques and may contribute to neurological complications seen in PWH.

## Introduction

Human immunodeficiency virus (HIV) infection has transitioned from a rapidly-progressing, fatal disease to a chronic infection with the intervention of combined antiretroviral therapy (ART) [1, 2]. ART successfully suppresses virus in the plasma and effectively increases the life expectancy of people with HIV (PWH) [3, 4]. Despite successful therapy, ART has limited penetrance in certain organs, such as the brain, leading to the formation of viral reservoirs and development of chronic neuroinflammation and neurological disorders [5]. In the pre-ART era, HIV-associated neurocognitive disorders were severe, since the onset of ART more mild forms predominate [6]. Despite suppression of viral replication, persistent neuroinflammation continues to perturb CNS homeostasis [7]. Although many studies have investigated HIV-associated neurological dysfunction, the molecular pathways where HIV viral proteins contribute to neurological impairment over the course of HIV infection are not fully understood.

The accessory *Negative Regulatory Factor* (*Nef)* gene is encoded by HIV-1, HIV-2 and the simian immunodeficiency virus (SIV) genomes [8]. HIV Nef is a 27–35 kDa protein expressed in the early stages of the viral life cycle and is the first viral protein produced in HIV-infected cells [8, 9]. NMR structural analysis shows that HIV Nef is composed of four major regions: a myristoylated N-terminal anchor domain, a proline-rich region, a conserved globular core structure, and a C-terminal flexible loop [8, 10]. The functional domains of HIV Nef act post-translationally to decrease the cell-surface expression of CD4, downregulate MHC-1 expression, modulate TCR signaling, increase HIV replication, and increase viral infectivity [11, 12]. *In vitro* studies indicate that Nef expression modulates the activation of the transcription factor NF-κB and IL-2 expression, implicating Nef in regulation of immune function [13]. Nef was initially considered an inhibitor of viral genome transcription [14], but studies have since shown that the *Nef* gene is crucial for maintenance of a robust, high viral production and is implicated in promoting disease progression to acquired immune deficiency syndrome (AIDS) [15]. Nef also increases the infectivity of HIV virions, as HIV-1 particles produced in the presence of *Nef* can be up to ten times more infectious than in the absence of *Nef gene* [16]. Exposure of human glial cells and neurons to HIV Nef demonstrated a neurotoxic effect to CNS resident cells [17], likely through an indirect effect of IP-10 or quinolinic acid [18]. HIV *Nef* deletion or mutation can diminish the neurotropism of HIV suggesting that Nef may play a role in viral seeding into the CNS [19]. Furthermore, rodent studies revealed that HIV Nef induces neurocognitive impairments in rats [20, 21]. Brain autopsy samples of PWH revealed that Nef was found in both macrophages and astrocytes. In addition, Nef-positive astrocytes were detected in six out of seven specimens from PWH who had moderate to severe cognitive impairment [22]. Interestingly, Nef was also found in frontal lobe neurons of patients with HIV associated dementia [23]. More interestingly, Nef expression in astrocytes associated with neuroinflammation causing neuronal damage and inducing spatial and recognition memory deficits [19, 21, 24, 25].

The role of HIV Nef in the perturbation of neurological impairment is not fully understood. Even in the presence of ART, neurocognitive deficits, including impairments in attention, memory processing and retrieval, are present in PWH. Here, we utilized a well-established SIV infection rhesus macaque model to further study and characterize Nef expression in the brain. Our results suggest that regardless of ART status, Nef is expressed in the brain of SIV infected macaques. In addition, by combining the state of the art RNAscope with IHC, we showed that Nef detection was not limited to the cells actively replicating the virus. Moreover, cell type specific staining revealed that in addition to astrocytes and Iba-1 positive microglia and macrophages, Nef was also present in neurons and its presence was associated with apoptosis. Altogether, our results suggest that Nef may be a formidable contributor of neurological impairment seen in PWH.

## Results

### Nef is expressed in the brain regions of SIV infected macaques

Tissue sections of frontal cortex, hippocampus and cerebellum from uninfected (SIV-, n = 3), SIV-infected animals with encephalitis (SIVE, n = 3), SIV-infected animals without encephalitis (SIV+ No ART, n = 3), and SIV-infected animals treated with ART (SIV+ART+, n = 3) were used to detect SIV Nef protein *in vivo* through immunohistochemistry. Viral, drug, and zoological information of animals used in the study are provided in Table 1.

**Table 1:** Viral, drug, and zoological information of animals used in the study.

Animal No.	Infection Status	CD8 Depletion	Survival (Days)	Age (years)	Term. Viral Load (Log10)	Brain pathology
C01	SIV-	N/A	N/A	3.28	N/A	N/A
C02	SIV-	N/A	N/A	3.41	N/A	N/A
C03	SIV-	N/A	N/A	2.27	N/A	N/A
A01	SIV+	Depleted	55	7.3	7.83	SIVE
A02	SIV+	Depleted	89	10.4	7.71	SIVE
A03	SIV+	Depleted	77	5.8	8.54	SIVE
A04	SIV+	Depleted	174	10.8	7.28	AIDS no E
A05	SIV+	Depleted	168	4.3	5.87	AIDS no E
A06	SIV+	Depleted	106	4.5	7.69	AIDS no E
A07	SIV+ART+	Depleted	120	6.2	2.34	Normal
A08	SIV+ART+	Depleted	118	6.7	2.66	Normal
A09	SIV+ART+	Depleted	118	6.1	2.66	Normal

N/A = Not Applicable, SIV- = Uninfected, SIV+ = SIV Infected, ART+ = ART treated, Term. = Terminal, SIVE = SIV Encephalitis, AIDS no E = Non-encephalitic

**Fig 1:**
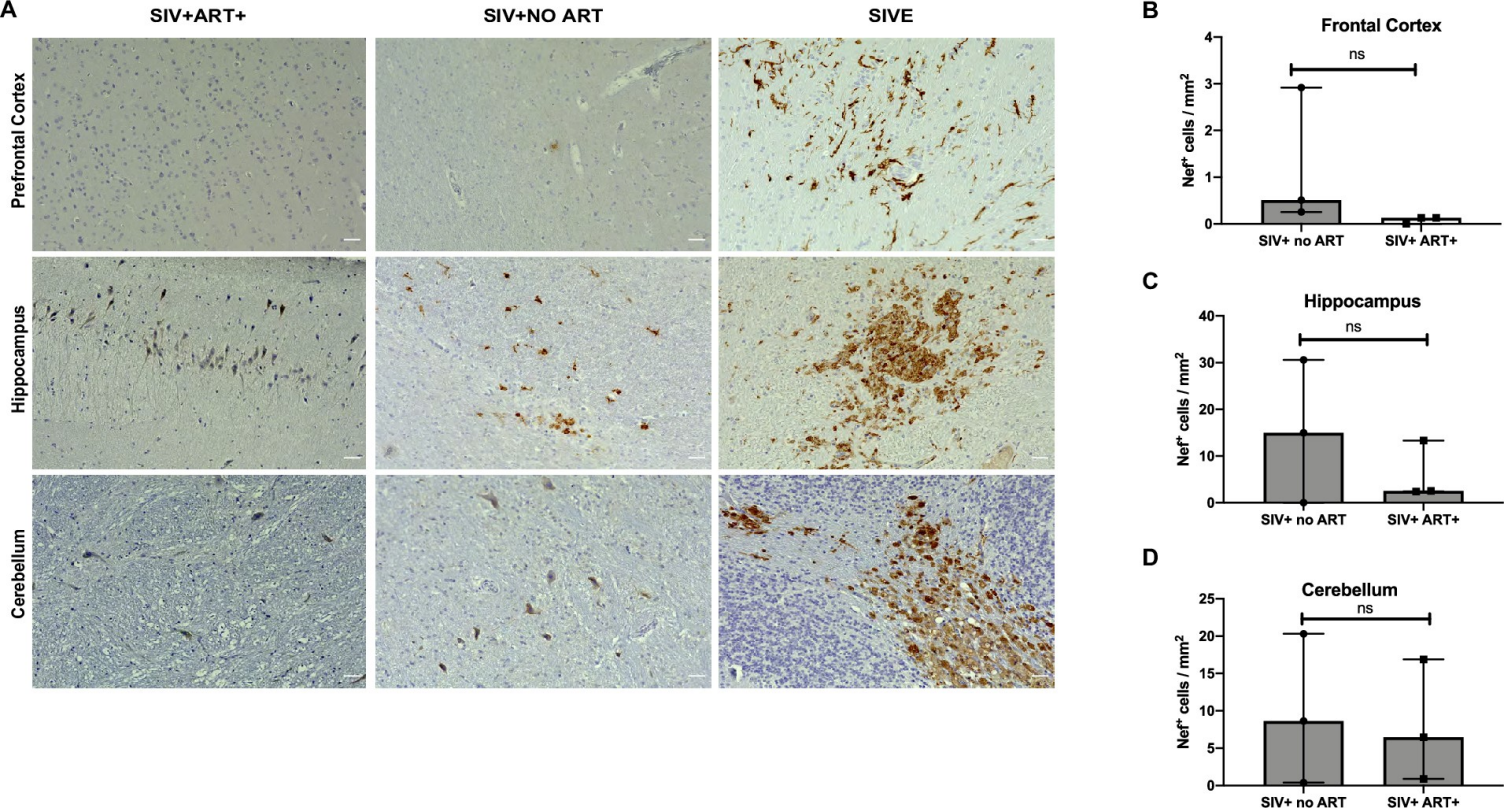
Immunohistochemical staining to detect SIV Nef in different brain sections of SIV-infected macaques. A. Representative images from paraffin embedded SIV-infected macaques brain tissue sections: frontal cortex, hippocampus and cerebellum in SIV+ ART +, SIV+ no ART and SIVE (no ART) were serial sectioned and stained for routine histological analysis and stained for SIV Nef. Representative images were taken at 20X magnification with a Keyence BZ-X700 microscope. B—D. Nef positive cells per mm^2^ of tissues from frontal cortex, hippocampus and cerebellum were quantified using BZ-X analyzer software (Keyence). (n = 3/per group). Five images were taken per condition per tissue. The average number per group was used for further statistical analysis. Comparison of means was determined by T-test. SIV+ ART+: SIV infected, receiving ART. SIV+ no ART: SIV infected, untreated, and SIVE: SIV infected with encephalitis. Scale bar represent 10 μM.

As expected, while SIV- (uninfected) tissues had no visible staining, SIV Nef staining was evident in the frontal cortex of animals with SIVE (Fig 1 and S1 Fig in S1 File). Interestingly, SIV Nef was very limited in cells from frontal cortex of SIV+ no ART and SIV+ART+ macaques (Fig 1A and 1B, S1 Fig in S1 File). On the other hand, hippocampal areas of SIVE animals showed lesions with high density of Nef staining and SIV+ no ART animals showed individually Nef positive cells. Interestingly, hippocampal regions of SIV+ART+ animals also showed Nef+ cells (Fig 1A and 1C, S2 Fig in S1 File). Cerebellar regions of SIV+ no ART and SIV+ART+ showed Nef+ cells in the molecular and Purkinje cell layers (Fig 1A and 1D, S3 Fig in S1 File). Nef positive lesions were found in the molecular layer and white matter of folium of the cerebellum of SIVE+ macaques. The SIVE animals were utilized as controls for active viral replication. For quantification purposes, and due to the current clinical presentation of HIV infection where encephalitis is rare, we did not include the lesions seen in SIVE in quantitative analysis and there was no significant difference between the number of Nef+ cells in SIV+ no ART and SIV+ ART+ in three different brain regions analyzed. These results suggest that SIV Nef is still expressed in the brain of SIV infected animals despite ART and reduced plasma viral loads.

### Cell type-specific Nef expression in hippocampus

**Fig 2:**
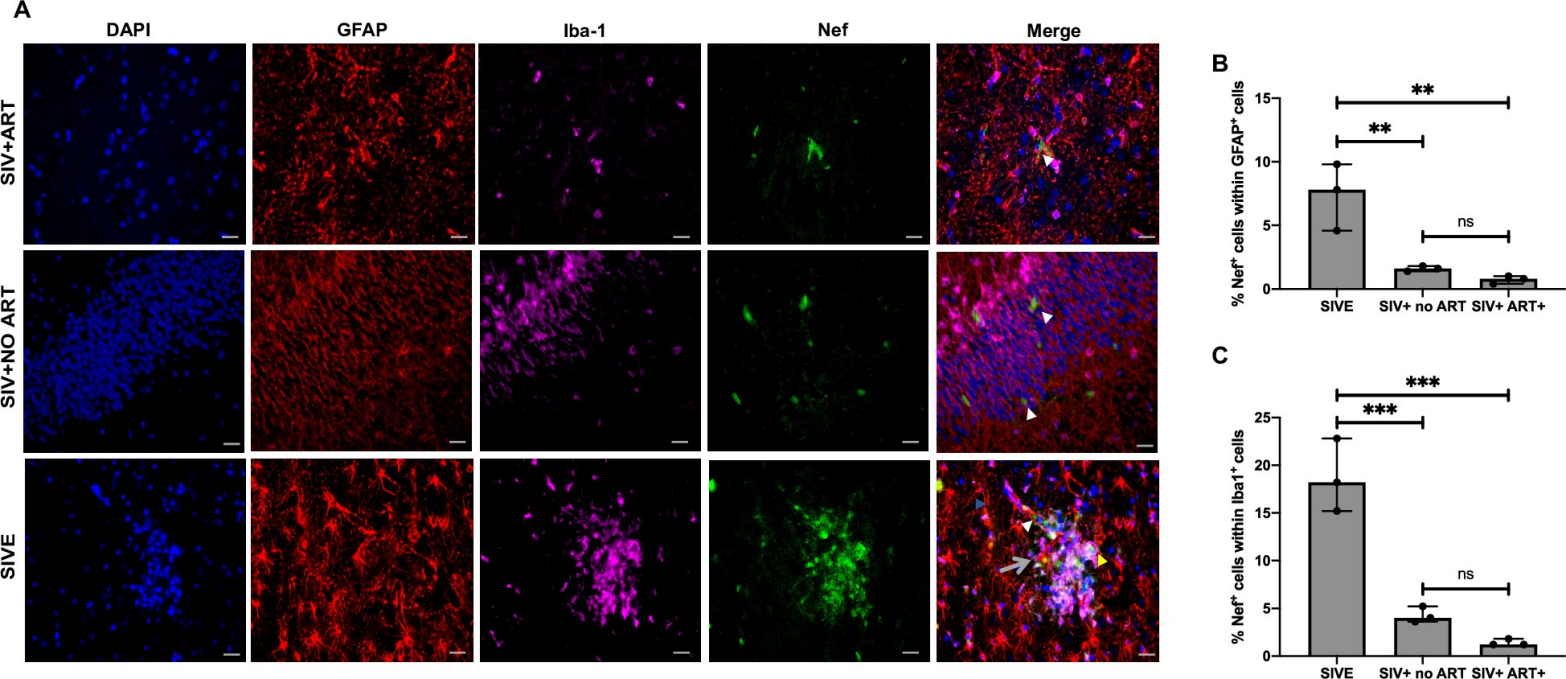
Cell type-specific Nef expression in hippocampus sections from SIV infected macaques. A. Paraffin embedded SIV-infected macaques brain hippocampus region were serial sectioned and stained for cells specific biomarkers GFAP (red), IBA-1 (pink) and Nef (green) using 4 color opal multiplex immunohistochemical assay staining (Perkin Elmer). Representative images were taken at 20X magnification with a Keyence BZ-X700 microscope (n = 3/ per group). B -C. Quantification of number of Nef+ cells within GFAP + cells (B) and Iba-1+ cells (C) per mm ^2^. Five images were taken per condition per tissue with 20x magnification. The average number per group was used for further statistical analysis. Comparison of means was determined by one-way ANOVA, followed by post-hoc Tukey HSD test **P<0.05, ***P<0.001. White Arrows: Nef+/GFAP-/ IBA-1- cells. Yellow arrows: Nef+/ IBA+ cells. Grey arrow with tail: NEF+/GFAP+ cells. Scale bar represent 10 μM.

SIV Nef protein co-localization with cell type specific markers was achieved through a Multiplexing immunofluorescent approach. Hippocampal regions of SIVE, SIV+ no ART, and SIV+ART+ animal cohorts were co-stained for GFAP, Iba-1 and SIV Nef (Fig 2, and S4 Fig in S1 File). Interestingly, hippocampal sections of SIV+ART+ animals showed SIV Nef+ cells that were negative for both GFAP and Iba-1 (Fig 2A, denoted by white arrows). The percent of Nef+GFAP+ cells was significantly different among SIVE (~8%), SIV+ no ART (~1.5%), and SIV+ ART + (~0.5%) animals (Fig 2B). Post-hoc analysis showed significantly less Nef+ GFAP+ cells in SIV+ no ART animals compared to SIVE and in SIV+ART+ compared to SIVE animals (P = 0.0084 and 0.0042). There was no significant difference between SIV+ no ART and SIV+ ART+ animals for Nef expression in GFAP+ cells. SIV Nef and Iba-1 overlap was also quantified (Fig 2C, denoted by yellow arrow). The percent Nef+Iba1+ cells were significantly different between SIVE (~19%), SIV+ no ART (~5%) and SIV+ART+ animals (~1%) (Fig 2C). Post-hoc analysis showed significantly less Nef+ Iba1+ cells in SIVE+ animals compared to SIV+ no ART and SIV+ART+ animals (P = 0.0006 and 0.0002). There was no significant difference between SIV+ no ART and SIV+ ART + animals for Nef expression in Iba-1 cells.

### Nef expression is not limited to the SIV RNA positive cells in hippocampus and cerebellum of SIV-infected animals

**Fig 3:**
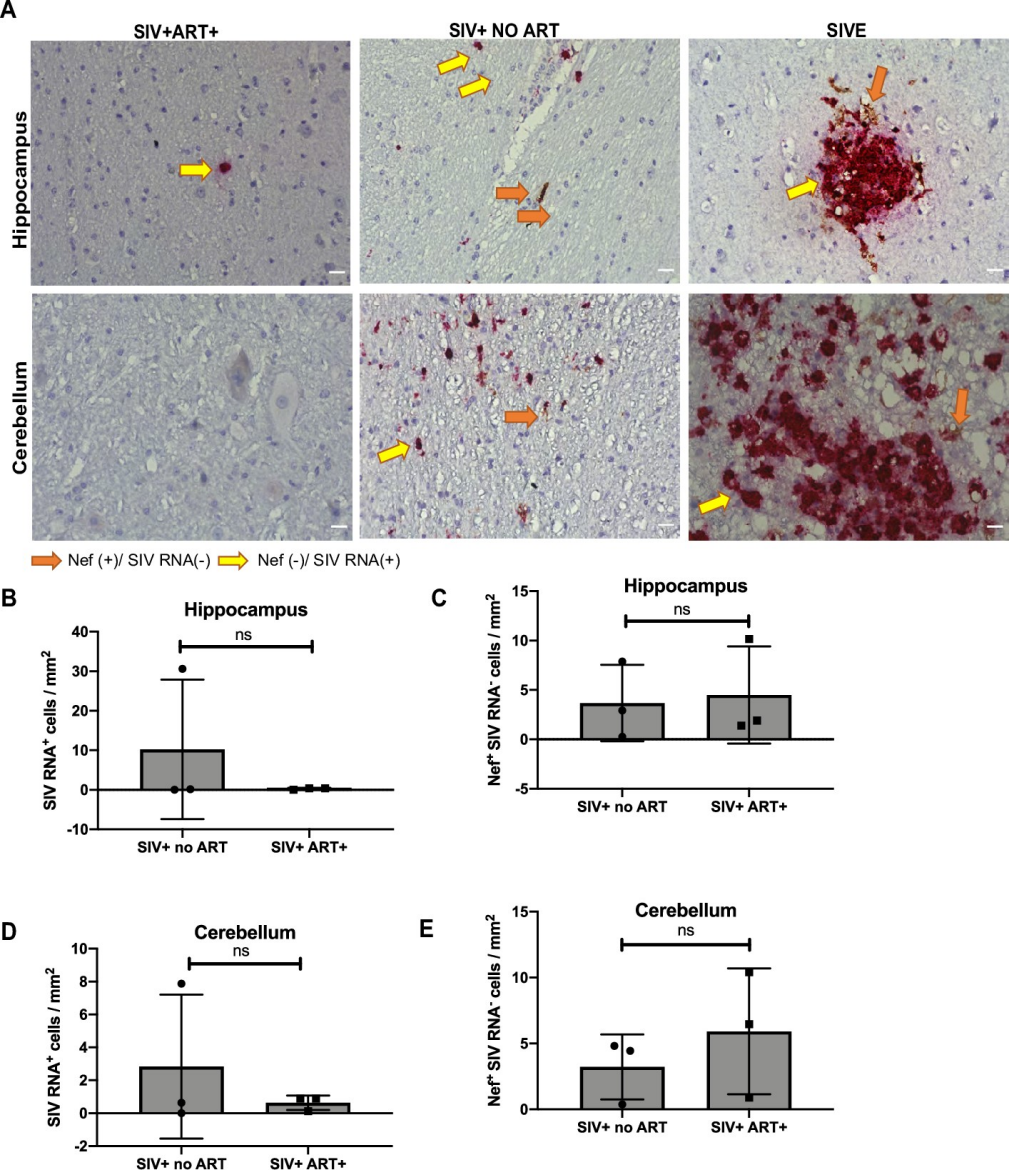
Nef + SIV RNA- cells are detected in different brain regions of SIV-infected animals. A. SIV RNA was visualized with RNAscope (Red) and Nef protein (Brown) markers by immunohistochemistry in the hippocampus and cerebellum of SIV-infected animals: SIV+ ART+, SIV+ no ART, and SIVE. Representative images were taken at 20X magnification with a Keyence BZ-X700 microscope. Five images were taken per condition per tissue and the average number per group was used for analysis. (n = 3/per group). B and D. Quantification analysis of SIV RNA+ cells in hippocampus and cerebellum region per mm^2^. C and E. Quantification analysis of number of Nef+/RNA- cells in the hippocampus and cerebellum region per mm^2^ using BZ-X analyzer software (Keyence). Yellow arrow: SIV RNA + and orange arrow: SIV RNA—and Nef+ cells. Scale bar represent 10 μM.

**Fig 4:**
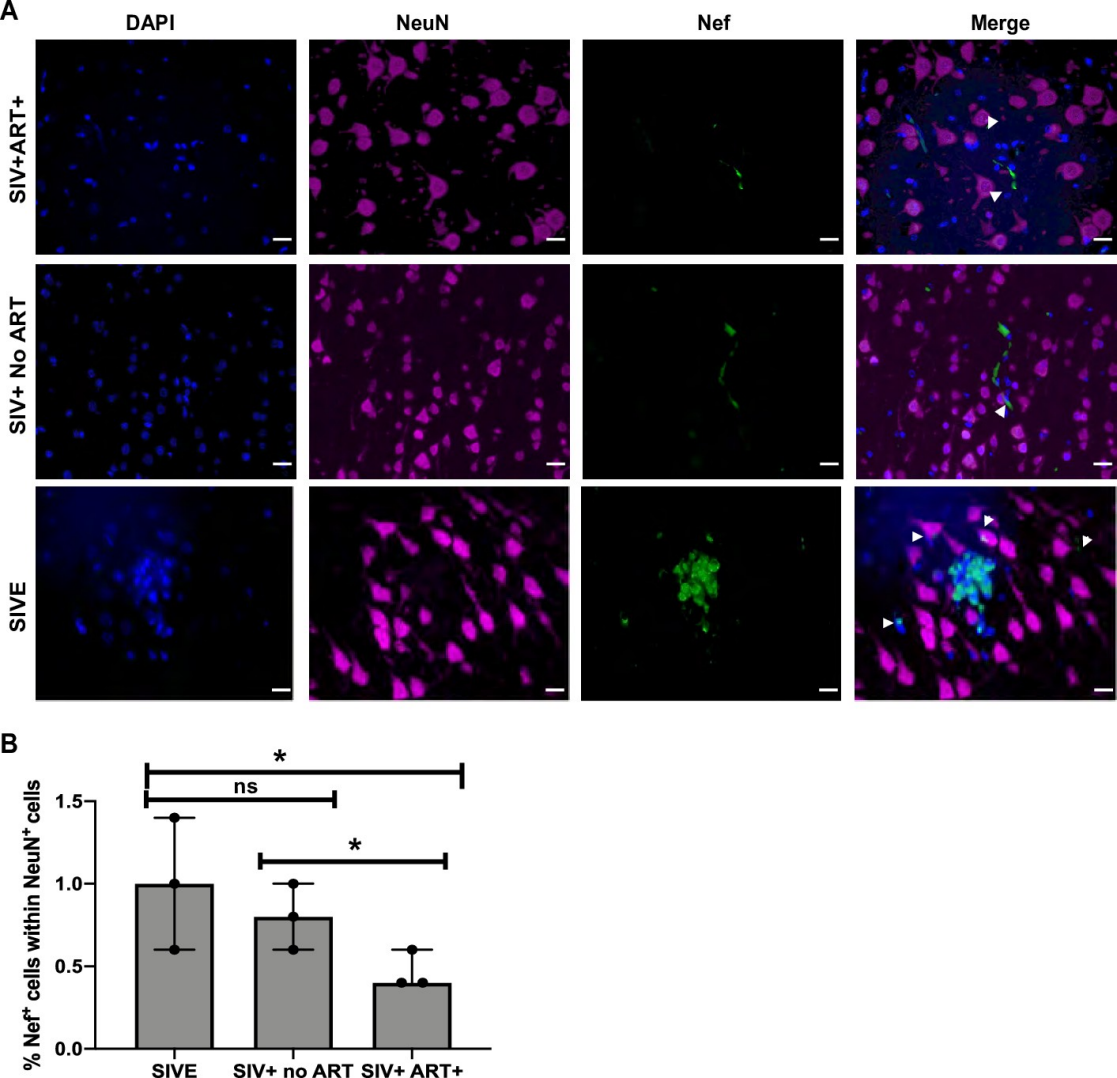
Detection of Nef in neurons from hippocampus of SIV- infected macaques. A. Hippocampus regions from paraffin embedded SIV-infected macaques brain samples were serial sectioned and stained for Neu-N (pink) and Nef (green) using opal multiplex immunohistochemical assay staining. Representative images were taken at 20X magnification with a Keyence BZ-X700 microscope. (n = 3/ per group). B. Quantification analysis of number of Nef+ within NeuN + cells in hippocampus region per mm^2^, five images were taken per condition per tissue and the average number per group was used (n = 3). Comparisons of means for significance was determined with T-test. Scale bar represent 10 μM. (P < 0.002).

In order to investigate if SIV Nef expression was limited to the SIV-infected cells, dual RNAscope and immunohistochemistry was performed to detect SIV viral RNA and SIV Nef protein in cerebellar and hippocampal regions of SIVE, SIV+ no ART, and SIV+ART+ groups. Interestingly, a population of SIV RNA- and Nef+ cells were observed in the hippocampus and cerebellum of SIVE and SIV+ no ART (Fig 3A, orange arrows, S5 and S6 Figs in S1 File). Quantification analysis of Nef+ SIV RNA- cells in hippocampus and cerebellum (Fig 3C and 3E) and RNA+ cells in hippocampus and cerebellum (Fig 3B and 3D) were also performed. Interestingly, while RNAscope revealed a significant decrease in SIV RNA expression in SIV+ART+ animals compared to SIV+ no ART, SIV RNA- Nef+ cells were undistinguishable. In order to further analyze if SIV RNA- Nef+ cells were neuronal phenotype, we also performed co-immunostaining of Nef protein with neuronal marker Neu-N. Hippocampal tissue sections were co-stained with SIV Nef and Neu-N, to determine possible overlap of Nef with neurons (Fig 4A and S7 Fig in S1 File). The observed colocalization of NeuN and Nef was evident in the perinuclear area in SIV+ART+, SIV+ no ART, and SIVE animals, suggesting the presence of Nef protein in neurons which are known to be not susceptible for SIV infection. Quantification of Nef+ and Neu-N+ cells suggested that co-expression of both proteins was about 1%, 0.8% and 0.4% in SIVE, SIV+ no ART and SIV+ART+ respectively (Fig 4B). Interestingly, while there was no significant difference between SIVE and SIV+ no ART, Nef+ neurons were significantly higher in SIV+ no ART group compared to the SIV+ ART+ animals and in SIVE group compared to SIV+ ART+ animals (P < 0.002).

### Neuronal Nef is associated with cleaved caspase-3 induction in hippocampus of SIV-infected animals

**Fig 5:**
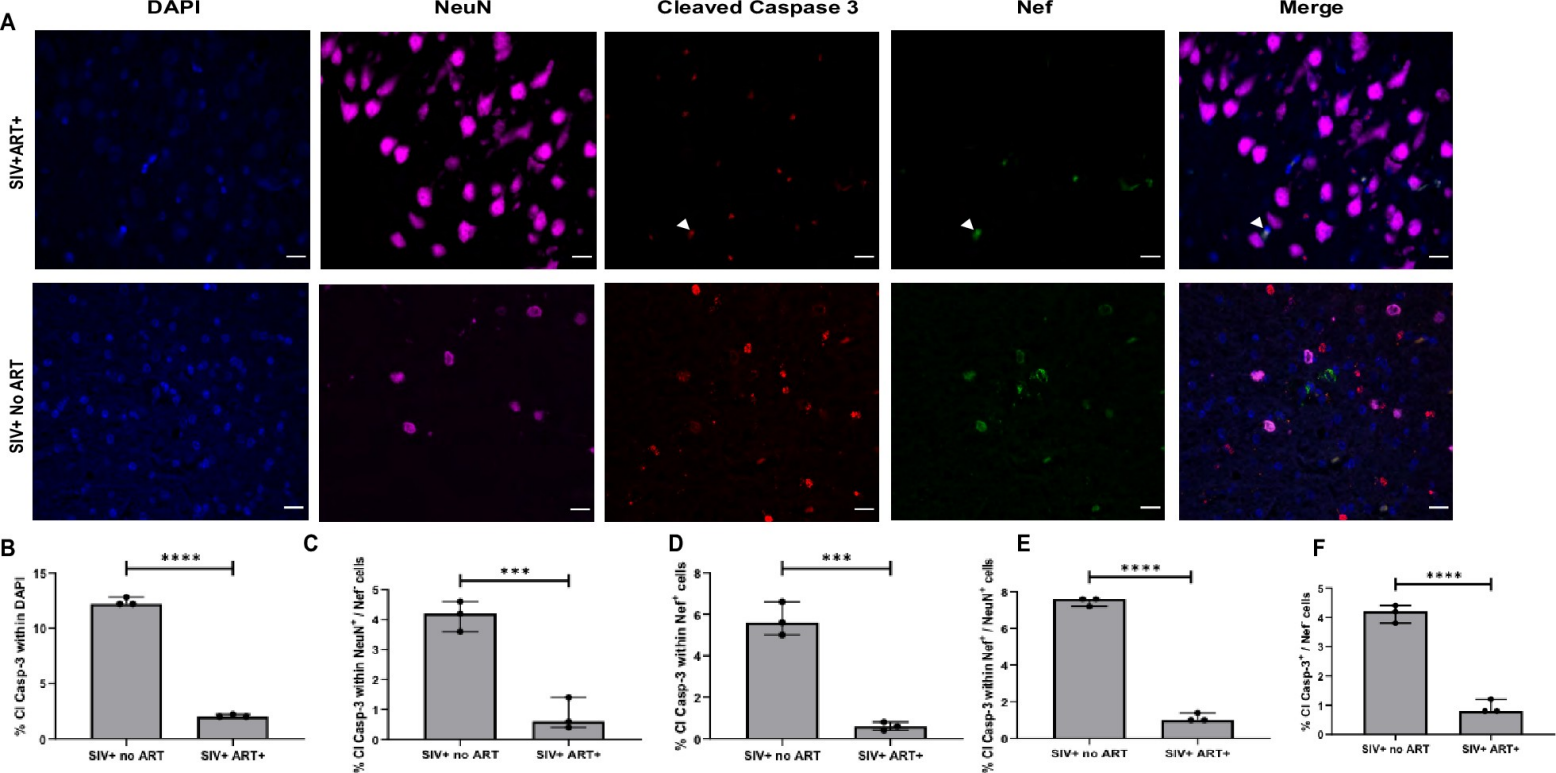
Multiplex analyses of Nef and cleaved caspase-3 expression in neurons. A. Hippocampus regions from SIV-infected macaques brain samples were serial sectioned and stained for cells specific biomarkers NeuN (pink), Cleaved caspase 3 (Red) and Nef (green) using 4 color opal multiplex immunohistochemical assay staining. (Perkin Elmer). Representative images were taken at 20X magnification with a Keyence BZ-X700 microscope. (n = 3 per group). B. Quantification analysis of number of cleaved caspase 3 positive cells per mm2. C. Quantification analysis of number of cleaved caspase 3 within NeuN+ and Nef- cells per mm2. D. Quantification analysis of number of % of cleaved caspase 3+ cells within Nef+ cells in the hippocampus region. E. Quantification analysis of number of cleaved caspase 3+ cells within Nef + and NeuN + cells in the hippocampus region. F. Quantification analysis of number of cleaved caspase 3+ cells within Nef—cells. Comparison of means was determined by T-test (***P<0.005, ****P<0.0001). Scale bar represent 10 μM.

In order to characterize neuroapoptosis with SIV infection in the brain and possible neuronal Nef association with neurotoxicity, we utilized the multiplex immunofluorescence to stain for Neu-N, cleaved caspase 3 and Nef protein (Fig 5 and S8 Fig in S1 File). Interestingly, hippocampal regions of SIVE, SIV+ no ART and SIV+ ART+ animals showed Nef+ lesions and Nef+ NeuN+ neurons that colocalized with cleaved caspase 3. The total number of cells that were positive for cleaved caspase 3 were determined per mm2. The percent of cleaved caspase 3 positive cells was significantly higher in SIV+ no ART (~12%) than SIV+ART+ (~ 2%) (P = 0.0001) (Fig 5B). Further quantification suggested that there was a significant difference in percent of cleaved caspase 3 within NeuN+ and Nef- cells between SIV+ no ART (~4%) and SIV+ART+ (~0.5%) (P <0.0001) (Fig 5C). The percentage of cleaved caspase 3 within Nef+ cells were also quantified. Approximately 6% and 1% of Nef + cells were positive for cleaved caspase 3 in SIV+ no ART and SIV+ ART+, respectively (P = 0.0002) (Fig 5D). Percentage of cleaved caspase 3 within Nef+ and NeuN+ cells were analyzed and approximately 7.6% and 1% of NeuN+ and Nef+ cells were also positive for cleaved caspase 3 in SIV+ no ART and SIV+ ART+ respectively (P< 0.0001) (Fig 5E). Further quantification analysis showed that 4% and 1% of cells were cleaved caspase 3+ and Nef–in SIV+ no ART and SIV+ ART+ groups (P<0.0001) (Fig 5F). Lesions seen in SIVE animals were not considered in quantification analysis due to the extensive neuronal loss at lesion sites. While neurons are not directly infected with the virus, they appear to acquire Nef protein and show proteolytic activation of late phase caspase 3 and induction of apoptosis.

## Discussion

HIV-1 was initially thought to target immune cells in the periphery including T lymphocytes, monocytes/macrophages, and dendritic cells. Although the mechanisms of HIV infiltration into the brain remain debatable, several studies have illustrated that CNS cells such as perivascular macrophages, microglia, and astrocytes are also susceptible to infection [26–29]. Microglia and perivascular macrophages serve as persistence and latent viral reservoirs, supporting productive HIV-1 infection [26–29]. Neurons are not infected with HIV, however, the virus targets neurons through viral proteins, such as Tat, gp120, and Vpr. Effects of HIV-1 Tat and gp120 expression in the brain have been extensively studied [30–32]. HIV Tat disrupts blood-brain barrier integrity and alters neuronal intrinsic excitability, affects calcium dysregulation, causes astrocytosis, and leads to the subsequent death of neurons [33]. Gp120 causes neuronal apoptosis, increases inflammatory cytokines, increases oxidative stress, and disrupts the blood-brain barrier [34]. Both Tat and gp120 appear to be well characterized causing serious neurotoxic consequences in HIV-1 infected brains [30, 33–35]. Despite gp120 and Tat and other viral proteins, little is known about the characteristics and potential impact of HIV-1 Nef in the development of HIV-associated neurological disorders. Accumulating evidence suggest that Nef is an important contributor to the development of HIV associated neurological disorders. It can cause significant decrease in metabolic activities and increase cell death rates [17]. *In vivo* expression of Nef leads to neuronal loss and neuroinflammatory response, including upregulation of IP-10. Interestingly, increased expression of IP-10 has been detected in the brains of HIV individuals with severe cognitive deficits [20]. Also, Nef induces the recruitment of peripheral immune cells such as macrophages into the CNS resulting in an increase in neuronal apoptosis and a neuroinflammatory profile [20, 21]. In our work, we have elucidated the expression of Nef protein in different brain regions of SIV-infected animals and correlated cell-type-specific Nef expression with neurotoxicity. The results of our study demonstrate that Nef is expressed in different brain regions; frontal cortex, hippocampus, and cerebellum. To our knowledge, this is the first *in vivo* study demonstrating Nef expression and characterizing different cell types expressing Nef in the brain of SIV-infected macaques. Furthermore, the data presented here demonstrate that neurons within the hippocampus are positive for Nef in macaques on ART indicating that despite ART therapy, Nef might be still present in the different cell types, including neurons in the brain. Our RNAscope analyses further revealed that within the hippocampus and cerebellum there are cells that are actively infected with SIV as evidenced by the detection of viral RNA in these regions and cells in the proximity of infected cells that are positive for Nef protein in which SIV RNA was not detected. These data suggest that Nef protein is most likely transported from the infected cells to the uninfected neuronal cells. One inherent limitation of this study is the sample size being small (n = 3 per group). Relatively small number of animals used in this study is due to the limited availability of large-scale primate tissues and its focus on the presence of HIV-1 Nef in different cell types in different brain regions of animals with different disease stages. In addition, our IHC studies with Iba-1 marker suggested that a significant number of Iba-1+ cells are also positive for Nef. Due to the shared lineage of microglia and macrophage/monocytes, Iba-1 only represents the overall lymphoid cells and does not necessarily suggest the lymphoid cell types. Whether Nef is preferentially expressed in certain lymphoid cells at different stage of the HIV CNS disease remains to be elucidated.

Several *in vivo* studies suggest that Nef protein can be transferred from infected to uninfected cells and is considered as circulating viral factor, contributing to HIV pathogenesis. Using CD4.Nef.GFP transgenic mice and chimeric SIV infected macaques, it was shown that intracellular Nef was transferred from T cells to the endothelium [36]. In addition, Nef protein was present in extracellular vesicles (EVs) isolated from SIV infected macaques and was able to transfer Nef into other recipient cells [37]. Furthermore, it was demonstrated that Nef was present in the sera from 27 out of 32 HIV-1 patients [38]. Several other independent groups have also detected HIV-Nef protein in EVs isolated from the plasma of HIV-infected patients [39–42]. Other studies demonstrated that intracellular Nef was detected in peripheral blood mononuclear cells (PBMCs) in HIV infected patients with and without antiretroviral therapy [36] and was detected in uninfected B cells of lymphoid follicles of infected individuals [43].

Several decades ago, *in vitro* and *in vivo* studies illustrated that cell-type-specific proteins are found in other cell types. [44, 45]. These observations were studied in greater depth, leading to the discovery of many mechanisms by which proteins traffic between cells. These include trogocytosis, an active protein transport mechanism with tight contacts between cells [46], and formation of nanotubes that extend between neighboring cells as 50–200 nm long F actin containing protrusions from the plasma membrane of one cell to another cells [47]. Another mechanism in which proteins and other biological material transferred between cells is the exosomal protein transport [48]. Exosomes are conserved intercellular communication strategies between cells in which they exchange materials such as nucleic acids, proteins, and lipids and are released from multivesicular bodies (MVBs) [48]. More recently, exosomes in CNS have gained attention since these vesicles serve as one means of physiologic and pathologic cargo transfer between neurons and glial cells. Oligodendrocytes and astrocytes have shown to release neuroprotective exosomes to support neuronal metabolism and homeostasis [49–52]. In addition to neuroprotection, exosomes are also known to transfer pathological proteins between cells, enhancing neurodegenerative disorders as evident by the neuronal release of α-Syn-containing exosomes that are uptaken by glial cells [53] and the spread of β-amyloid peptide and tau protein to the extracellular space [54–56]. HIV Nef may also utilize multiple mechanisms to get transported from infected to the uninfected cells. Indeed, Nef was shown to be transferred to the bystander T cells by trogocytosis [57]. Nef is also shown to induce TNT formation [58, 59], and can be transferred from infected macrophages to the B lymphocytes [60], macrophages to T cells [59], Nef-expressing T cells to hepatocytic cells [61], and T cells and monocytes to human coronary arterial endothelial cells [36]. Accumulating evidence suggests that Nef -associated EVs from Nef expressing donor cells have damaging effects on recipient cells [37, 41, 62–66]. We also recently reported that Nef is released from infected astrocytes in association with EVs that are taken up by primary neurons [67]. These Nef carrying EVs suppressed functional neuronal action potential as it was assessed by multi-electrode array studies and caused neurotoxicity. Here, in line with the previous studies, our data suggest that the presence of Nef in neurons is associated with induction of cleaved caspase 3, indicative of late apoptosis and neurotoxicity.

HIV infection in the brain manifests itself at different levels in neuronal activities leading to a wide spectrum of neurological disorders ranging from mild cognitive deficiencies to motor functions and behavior abnormalities. PWH receiving ART have reduced risk of neurocognitive impairment but still are at a greater incidence of neurological impairments compared to the uninfected population [68]. Overall in this study, we characterized Nef expression in different brain regions of SIV-infected macaques. Interestingly, our data suggest that Nef is expressed in the hippocampus and cerebellum of SIV-infected animals despite treatment with ART. We further shed light on the different cell types positive for Nef protein including neurons that are undergoing apoptosis in ART-treated animals. Expression and secretion of viral proteins are proportional to viral load [69] but are not necessarily eliminated by ART, as illustrated with Nef expression and its association with apoptosis. Overall, our study highlights the possible role of HIV secretory proteins including Nef in HIV CNS disease seen in PWH.

## Material and methods

### Animals used in the study and ethical statement

Twelve male, Indian rhesus macaques (Macaca mulatta) were used in this study (Table 1). Three rhesus macaques (C01-C03, SIV-) served as uninfected controls. Nine animals (A01-A09, SIV+) were injected intravenously with SIVmac251 viral swarm (5ng p27; Tulane National Primate Research Center’s (TNPRC) Viral Core). A subcutaneously a dose of 5mg/kg of anti-CD8 antibody was administered at 6 days post-infection (dpi) and 5mg/kg of the antibody intravenously at 8 and 12 dpi (Nonhuman Primate Reagent Resource) for CD8 depletion. The SIV-infected (SIV+) animals were sacrificed according to humane endpoints consistent with the recommendations of the American Veterinary Medical Association (AVMA) Guidelines for the Euthanasia of Animals. The development of simian AIDS was determined post-mortem by the presence of Pneumocysti carinii-associated interstitial pneumonia, Mycobacterium avium-associated granulomatous enteritis, hepatitis, lymphadenitis, and/ or adenovirus infection of surface enterocytes in both small and large intestines. SIVE was defined by the presence of multinucleated giant cells, accumulation of macrophages in the CNS, and productive viral infection [51]. It is important to note that SIVE animals did not receive ART. Three animals (A10-A12, SIV+ART) received a triple ART regimen: Raltegravir (22 mg/kg oral twice daily, Merck), Tenofovir (30 mg/kg subcutaneous once daily, Gilead), and Emtricitabine (10 mg/kg subcutaneous once daily, Gilead) at 21 dpi until the timed sacrificed at 118–120 dpi. Animals were anesthetized with ketamine-HCL and euthanized by intravenous pentobarbital overdose. Animals used in the study were housed at the TNPRC. All animals used in this study were handled in strict accordance with American Association for Accreditation of Laboratory Animal Care with the approval of the Institutional Animal Care and Use Committee of Tulane University. Monkeys were housed in pairs to promote the psychological well-being of nonhuman primates. Enrichment included manipulable items in cage (durable and destructible objects), perches or swings, various food supplements (fruit, vegetables, primate treats), foraging or task-oriented feeding methods and human interaction with caretakers and research staff as dictated by The United States Animal Welfare Act. Enrichment devices are rotated on a weekly basis and include toys, mirrors, radios, TV/VCRs, foraging boards, and a variety of complex foraging devices. Animals were fed a normal monkey chow. Animal care staff monitored the health and well-being of the animals on a daily basis with physical examinations performed weekly. All possible measures were taken to minimize discomfort of the animals. Anesthesia and analgesics were used and administered under the direction of a licensed veterinarian. All procedures were performed under ketamine or telazol anesthesia. Analgesics such as buprenorphine and lidocaine were used preemptively and following each potentially painful procedure. All animals were euthanized according to humane endpoints consistent with the recommendations of the American Veterinary Medical Association (AVMA) Guidelines for the Euthanasia of Animals. Endpoint criteria for euthanasia included the following: weight loss >15% in 2 weeks or >30% in 2 months, documented opportunistic infection, persistent anorexia >3 days without explicable cause, severe, intractable diarrhea, progressive neurologic signs, significant cardiac and/or pulmonary signs or any other serious illness.

### Immunohistochemistry (IHC)

For histologic examination, frontal cortex, hippocampus and cerebellum regions of brains from uninfected animals (SIV-), SIV-infected animals with encephalitis, without receiving ART (SIVE), SIV-infected animals without encephalitis (SIV+), and SIV-infected animals treated with ART (SIV+ART+) were fixed in 10% formalin and embedded in paraffin. Paraffin-embedded tissues were cut in five-micron thick sections and analyzed for protein expression by IHC. Sections were deparaffinized in xylene and hydrated in 100%, 90%, and 75% of ethanol. Sections were heat-treated at 95°C for 20 min in an antigen unmasking solution (Vector, H-3300) for heat-induced antigen retrieval, cooled to room temperature, and washed in Phosphate-Buffered Saline (PBS) (Invitrogen). The sections were incubated for 5 minutes in Dual Endogenous enzyme block (Dako) and 30 minutes in Protein block (Dako). SIV-Nef primary antibody (1:1500), as detailed in Table 2, was prepared in antibody diluent (Dako), and the sections were incubated for overnight at 4°C. Slides were washed three times with DPBS. Sections were incubated in polymer-based HRP-conjugated anti-mouse Dako EnVision system and developed with 3,3′-diaminobenzidine (DAB) chromogen. All paraffin-embedded sections were counterstained with Harris hematoxylin (Sigma). Controls included uninfected animals. Images were obtained with a Keyence, BZ-X710 microscope. Five images were taken per tissue per condition with 20x magnification and SIV-Nef staining was quantified using BZ-X analyzer software (Keyence). The average number per group was used for further statistical analysis.

**Table 2:** Primary and secondary antibodies used in the study.

Antibody	Source	Concentration	Secondary Antibody	Source	Fluorophore
Mouse anti-SIV-Nef	Thermofischer MA1-71522	1:1500	polymer-based HRP-conjugated anti-mouse	Dako EnVision system/ Agilent	N/A
Mouse anti-SIV-Nef	Thermofischer MA1-71522	1:1500	Opal polymer HRP Ms+Rb	Perkin Elmer, Opal™ 4-Color IHC Kit NEL810001KT	Opal 520
Mouse anti Neu-N	Millipore MAB377	1:1000	Opal polymer HRP Ms+Rb	Perkin Elmer, Opal™ 4-Color IHC Kit NEL810001KT	Opal 690
Rabbit Anti IBA-1	Wako 019–19741	1:1000	Opal polymer HRP Ms+Rb	Perkin Elmer, Opal™ 4-Color IHC Kit NEL810001KT	Opal 690
Rabbit Anti- GFAP	Proteintech 23935-1-AP	1:2000	Opal polymer HRP Ms+Rb	Perkin Elmer, Opal™ 4-Color IHC Kit NEL810001KT	Opal 570
Rabbit Anti- Cleaved Caspase 3	Calbiochem 2923436)	1:1500	Opal polymer HRP Ms+Rb	Perkin Elmer, Opal™ 4-Color IHC Kit NEL810001KT	Opal 690

### Quantitative RNAscope in situ hybridization

SIV viral RNA was visualized using RNAscope on paraffin embedded tissues from hippocampus and cerebellum of SIVE, SIV+, and SIV+ART+ according to manufacturer’s protocols, using the SIVmac239 probe and RNAscope 2.5 HD Red Chromogenic Assay (Advanced Cellular Diagnostics, Newark, CA, cat #322360). Uninfected animals served as a negative control. Double labeling for SIV RNAscope followed by IHC staining for Nef protein, as previously reported [70, 71]. Slides were stained for anti-SIV-Nef. Sections were incubated in Polymer-based HRP-conjugated anti-mouse Dako EnVision system and developed with DAB chromogen. Sections were counterstained with hematoxylin, dried at 60°C, and mounted. Images were obtained with Keyence, BZ-X710 microscope and analyzed for colocalization of SIV viral RNA and SIV Nef protein. Five images were taken per tissue with 20x magnification and SIV-Nef staining was quantified using BZ-X analyzer software (Keyence). The average number per group was used for further statistical analysis.

### Opal multiplex immunohistochemistry

Sections from hippocampus region of SIVE, SIV+, and SIV+ART+ macaques were deparaffinized and rehydrated through ethanol. Slides were treated for antigen unmasking using an AR6 buffer (Perkin Elmer) according to manufacturer’s guidelines (Microwave: 45 seconds, 100% power; 15 min, 20% power). Slides were allowed to cool for 30 min at room temperature and washed with 1X PBS. Slides were incubated for 10 min in blocking reagent, rinsed with 1X PBS, and incubated with the primary antibodies for one hour, as detailed in Table 2.

Slides were incubated with polymer HRP Ms + Rb for 10 min at room temperature. Opal dyes were diluted in amplification reagent (1:100) and slides were incubated in dyes for 10 minutes. Following development, slides were re-treated with AR6 buffer (Microwave: 45 seconds, 100% power; 15 min, 20% power) to strip slides of antibody complexes and allow consecutive staining without concern for antibody isotypes. Detection for each marker was completed before application of the next antibody. Slides were counterstained with spectral DAPI (Perkin Elmer) for 5 min at room temperature and washed with 1XPBST buffer and water. Coverslip slides were mounted using ProLong® Diamond Antifade Mountant (Thermofisher). All reagents are from Perkin Elmer, Opal™ 4-Color IHC Kit NEL810001KT. Images were obtained with Keyence, BZ-X710 microscope. Five images were taken per tissue with 20x magnification and SIV-Nef staining was quantified using BZ-X analyzer software (Keyence). The average number per group was used for further statistical analysis.

### Statistical analysis

All statistics and graphical representations were done using GraphPad Prism version 8.0. Results are expressed as Mean +/- SEM. T-test was used in comparison of two unpaired groups. One-way ANOVA was used to compare three non-paired groups followed by a post-hoc Tukey HSD test. P value < 0.05 was considered significant.

## Supporting information

S1 File (PDF)
