# Supplementary material for: Characterization of Nef expression in different brain regions of SIV-infected macaques
Source: PLoS One. 2020 Nov 2;15(11):e0241667. doi: 10.1371/journal.pone.0241667 (PMC7605674; doi:10.1371/journal.pone.0241667)
Supplement: S1 File — (PDF) [file pone.0241667.s001.pdf]

Characterization of Nef expression in different brain regions of SIV-infected macaques

Shadan S. Yarandi[1], Jake A. Robinson[1], Sarah Vakili[1], Martina Donadoni[1], Tricia H. Burdo[1*], Ilker K. Sariyer[1*]

1. Department of Neuroscience, Center for Neurovirology, Lewis Katz School of Medicine at Temple University, Philadelphia, PA 19140.

Supplementary Additional Data

*Co-corresponding Authors
Dr. Ilker K. Sariyer
Associate Professor
Department of Neuroscience, Center for Neurovirology
Lewis Katz School of Medicine at Temple University
3500 North Broad Street
Medical Education and Research Building 753
7th Floor, Philadelphia, PA 19140
Tel: 215-707-6337
Email: isariyer@temple.edu

Dr. Tricia H. Burdo
Associate Professor, Associate Chair for Education
Department of Neuroscience, Center for Neurovirology
Lewis Katz School of Medicine at Temple University
3500 North Broad Street
Medical Education and Research Building 755
7th Floor, Philadelphia, PA 19140
Tel: 215-707-1618
Email: burdot@temple.edu

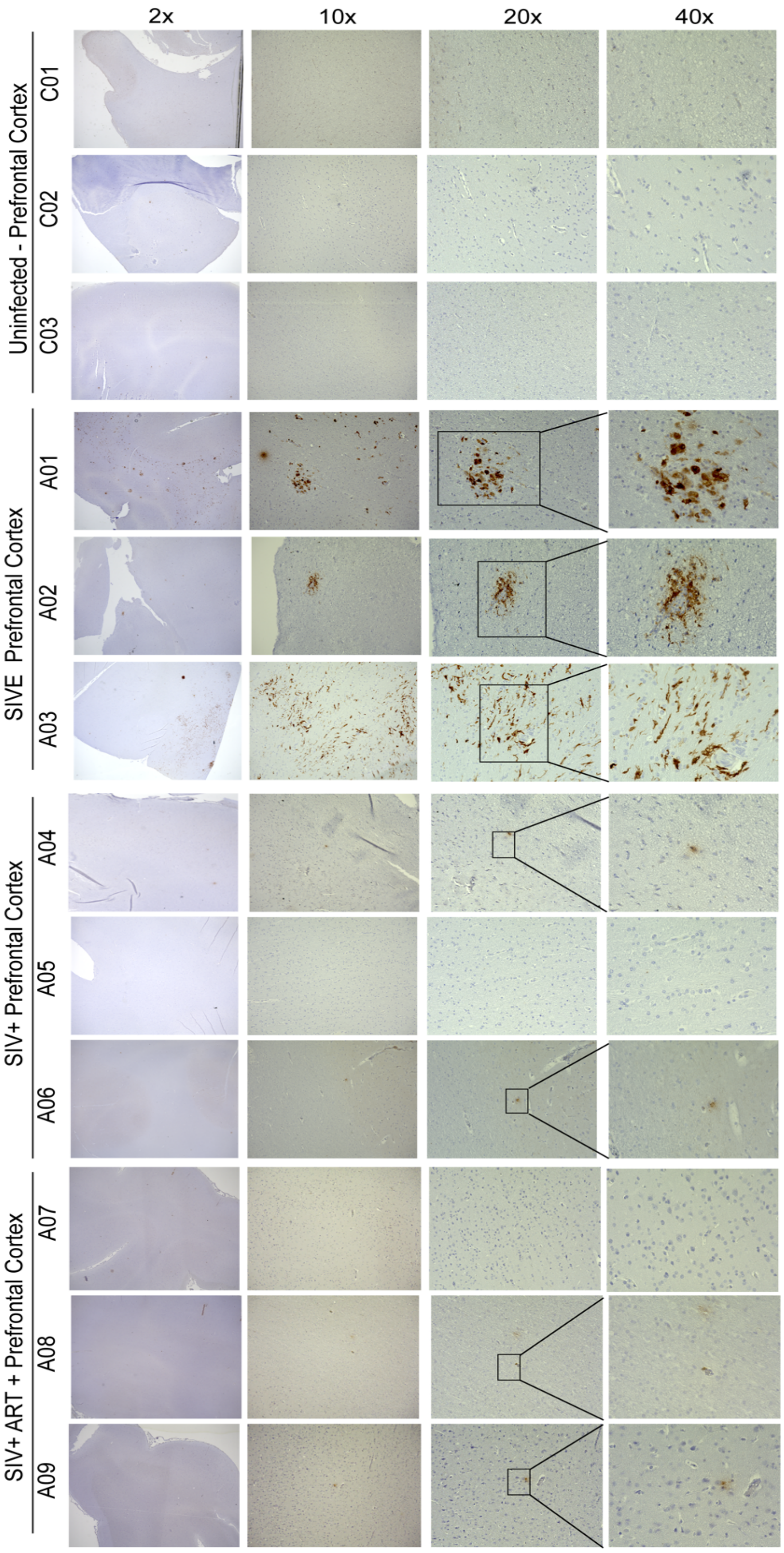

Supplementary figure 1. Immunohistochemical staining to detect SIV Nef in prefrontal cortex section of uninfected and SIV-infected macaques. Paraffin embedded uninfected and SIV-infected macaques brain tissue sections from prefrontal cortex of SIV+ ART +, SIV+ and SIVE were serial sectioned and analyzed for routine histological analysis and stained for SIV Nef. Images were taken at 2, 10, 20 and 40X magnification with a Keyence BZ-X700 microscope. (n=3/ per group).

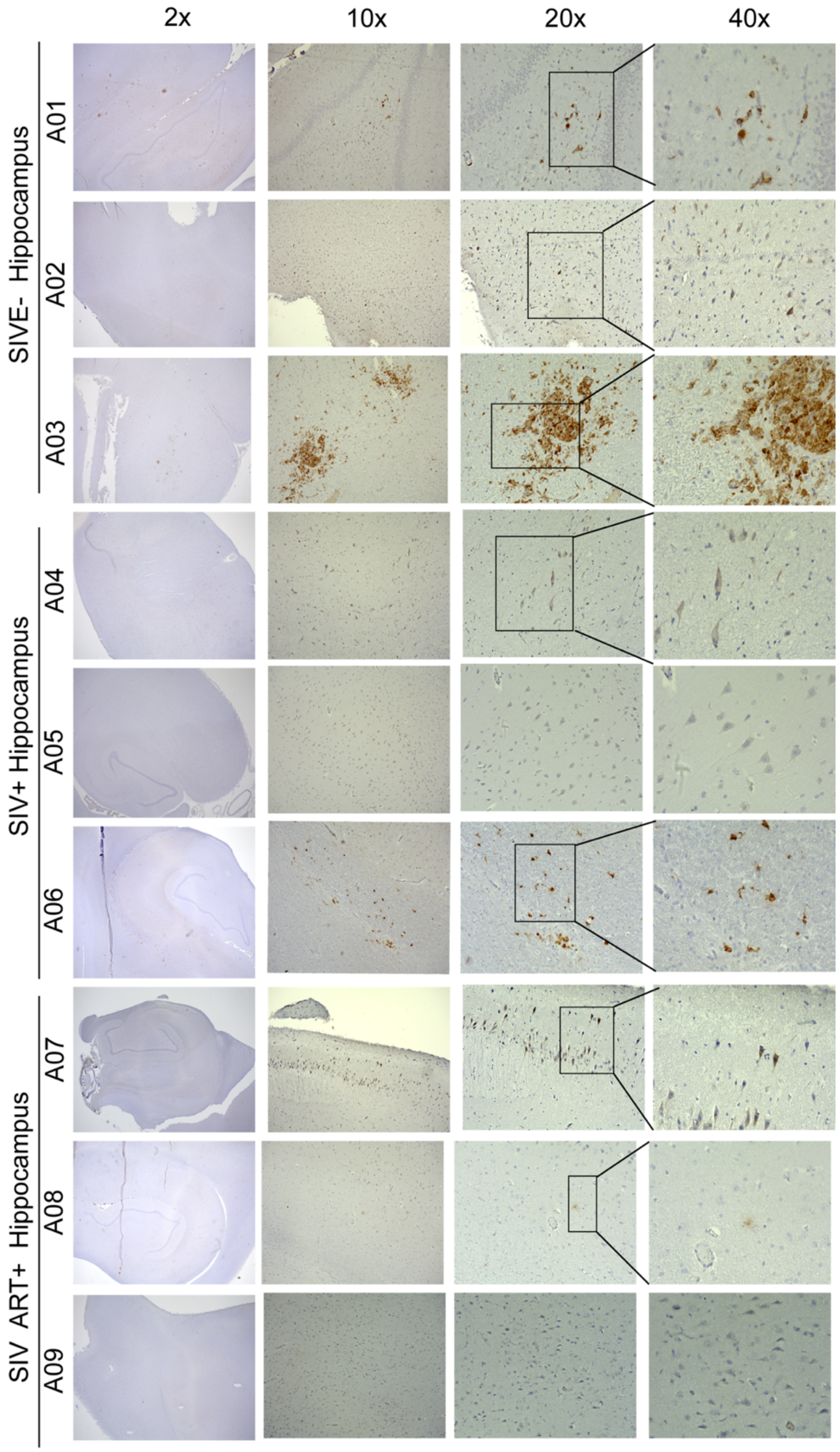

Supplementary figure 2. Immunohistochemical staining to detect SIV Nef in hippocampus region of uninfected and SIV-infected macaques. Paraffin embedded brain tissue sections from hippocampus regions of SIV+ ART +, SIV+ and SIVE were serial sectioned and analyzed by routine histological analysis and stained for SIV Nef. Images were taken at 2, 10, 20 and 40X magnification with a Keyence BZ-X700 microscope. N=3 per group.

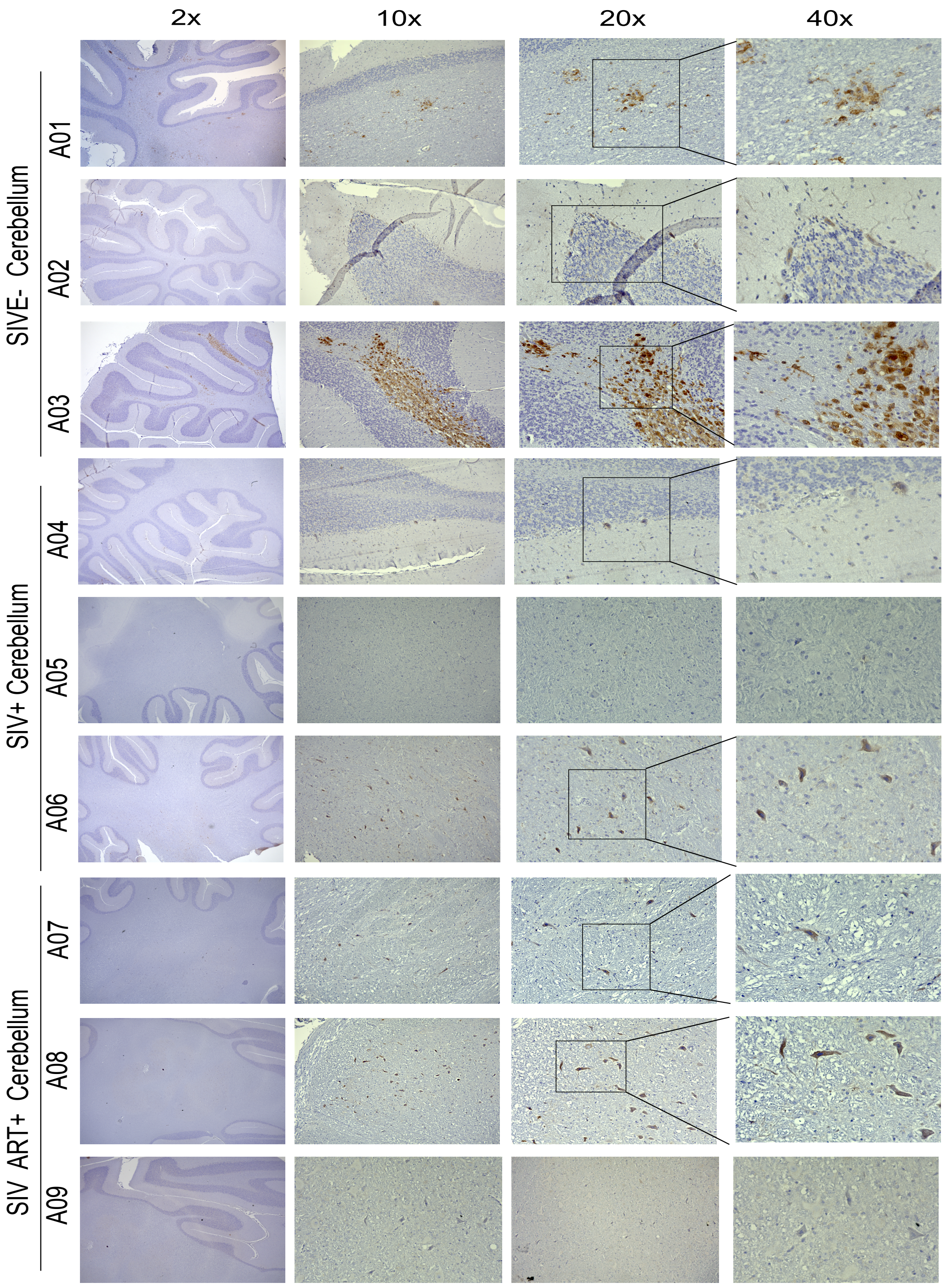

Supplementary figure 3. Immunohistochemical staining to detect SIV Nef in cerebellum region of SIV-infected macaques. Paraffin embedded brain tissue sections from cerebellum regions of SIV+ ART +, SIV+ and SIVE were serial sectioned and analyzed for routine histological analysis and stained for SIV Nef. Images were taken at 2, 10, 20 and 40X magnification with a Keyence BZ-X700 microscope. N=3 per group.

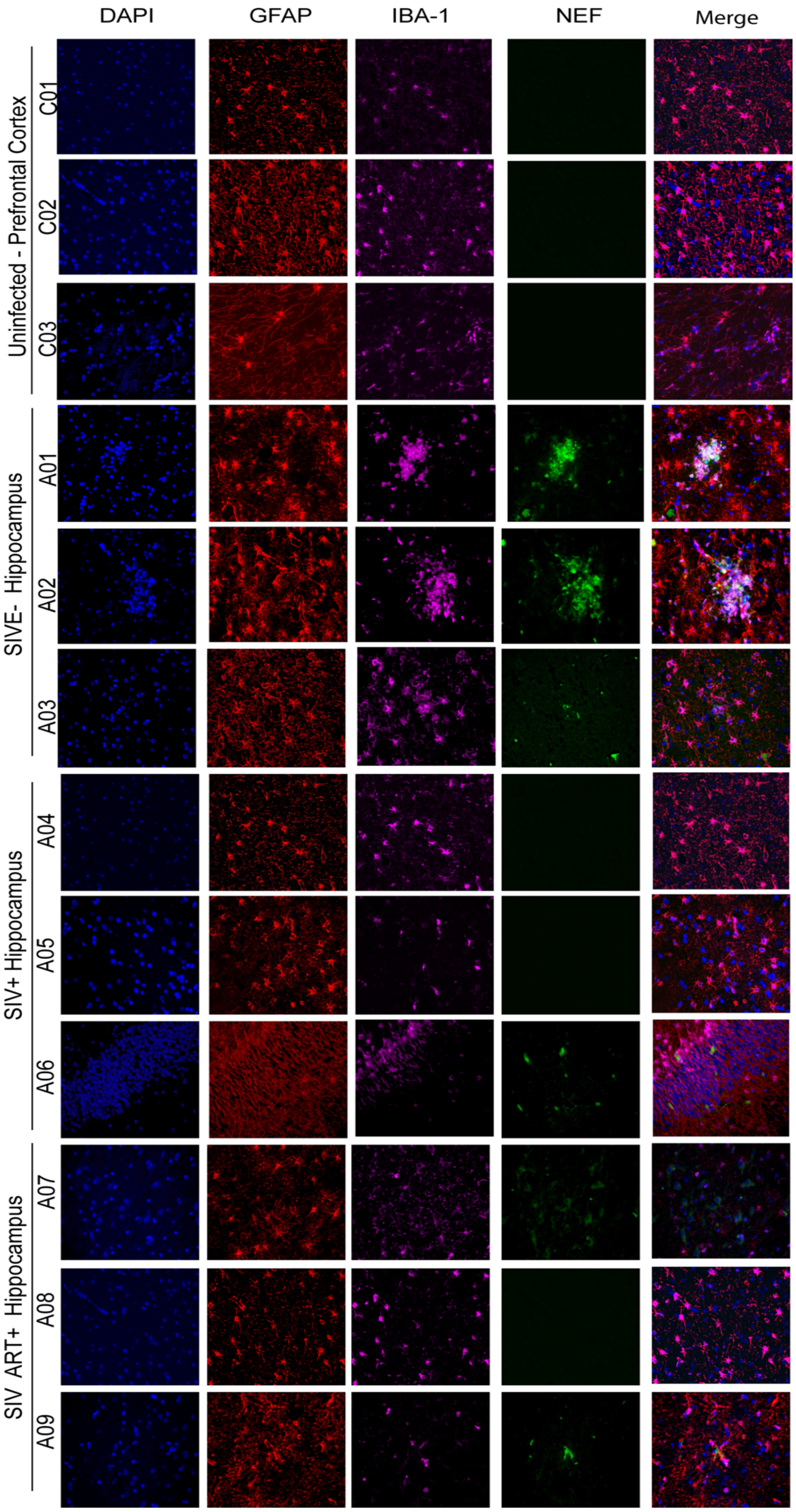

Supplementary figure 4. Cell type-specific Nef expression in hippocampus sections from SIV infected macaques. Paraffin embedded uninfected macaques brain prefrontal cortex and SIV-infected macaques brain hippocampus regions were serial sectioned and stained for cells specific biomarkers GFAP (red), IBA-1 (pink) and Nef (green) using 4 color opal multiplex immunohistochemical assay staining (Perkin Elmer). Images were taken at 20X magnification with a Keyence BZ-X700 microscope (n=3/ per group).

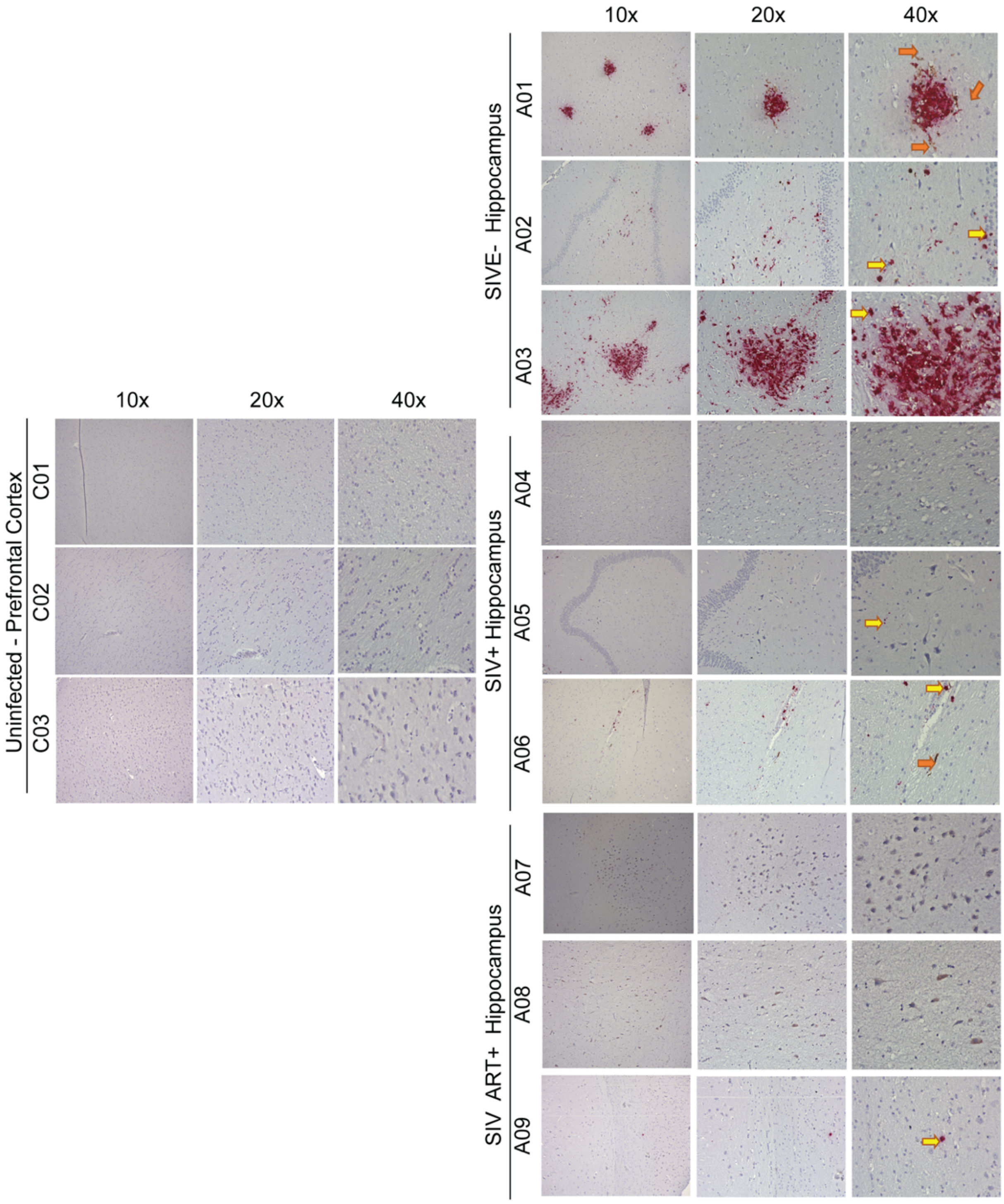

Supplementary figure 5. Nef + SIV RNA- cells are detected in hippocampus regions of SIV-infected macaques. SIV RNA was visualized with RNAscope (Red) and Nef protein (Brown) markers by immunohistochemistry in the prefrontal cortex of uninfected animals and hippocampus of SIV-infected animals: SIV+ ART+, SIV+ no ART, and SIVE. Images were taken at 10, 20 and 40X magnification with a Keyence BZ-X700 microscope. (n=3/per group).

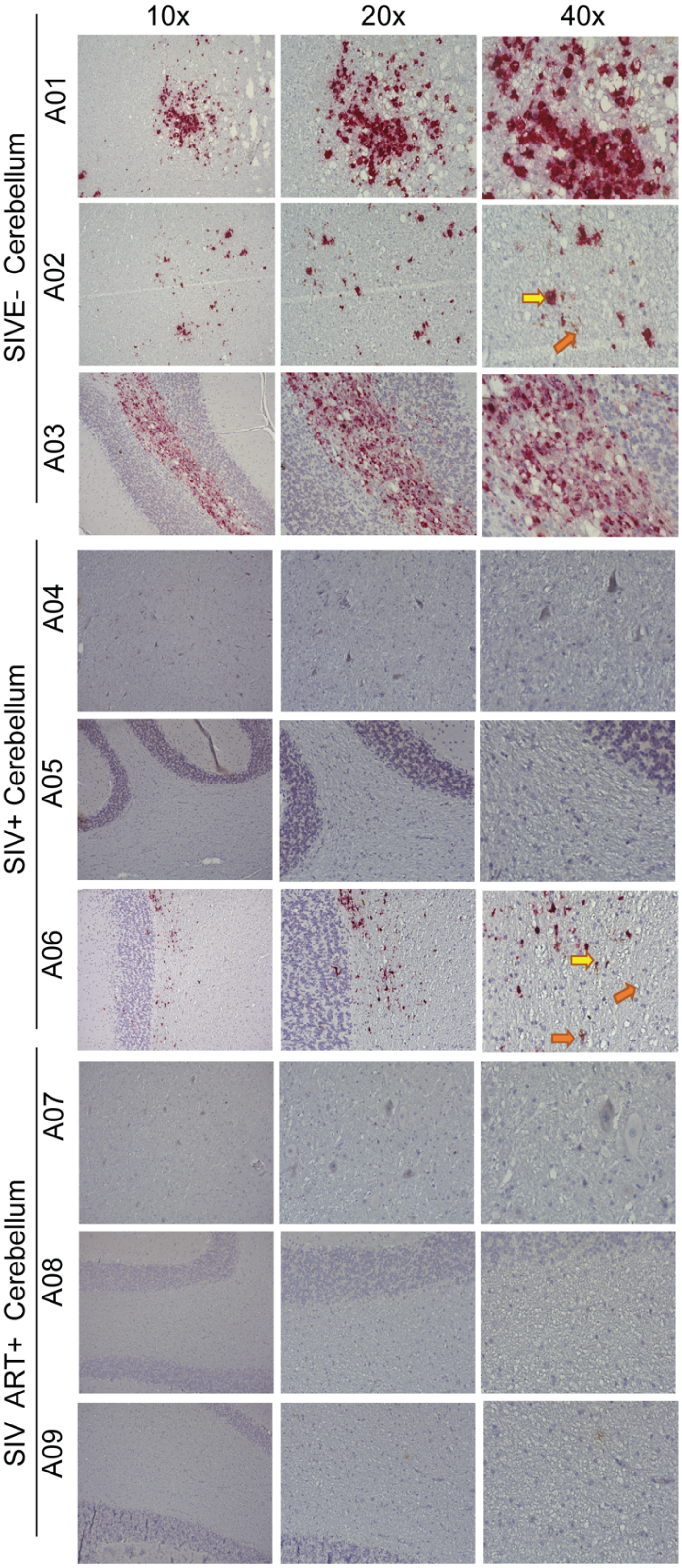

Supplementary figure 6. Nef + SIV RNA- cells are detected in cerebellum regions of SIV-infected animals. SIV RNA was visualized with RNAscope (Red) and Nef protein (Brown) markers by immunohistochemistry in the cerebellum of SIV-infected animals: SIV+ ART+, SIV+ no ART, and SIVE. Images were taken at 10, 20 and 40X magnification with a Keyence BZ-X700 microscope. (n=3/per group)

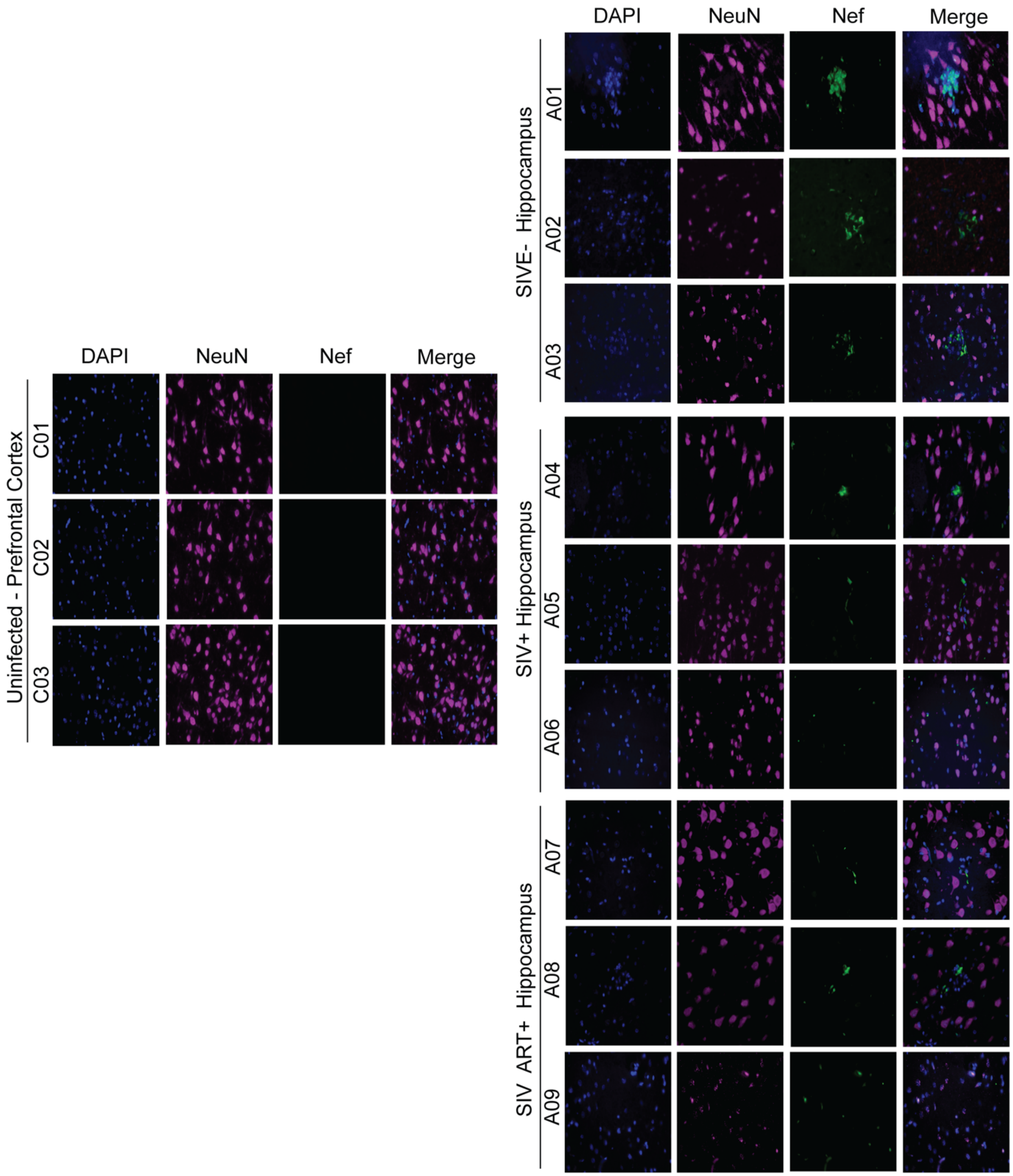

Supplementary figure 7. Detection of Nef in neurons from hippocampus of SIV- infected macaques. Pre-frontal cortex and hippocampus regions from paraffin embedded uninfected animals and SIV-infected macaques brain samples, respectively, were serial sectioned and stained for Neu-N (pink) and Nef (green) using opal multiplex immunohistochemical assay staining. Images were taken at 20X magnification with a Keyence BZ-X700 microscope. (n=3/ per group).

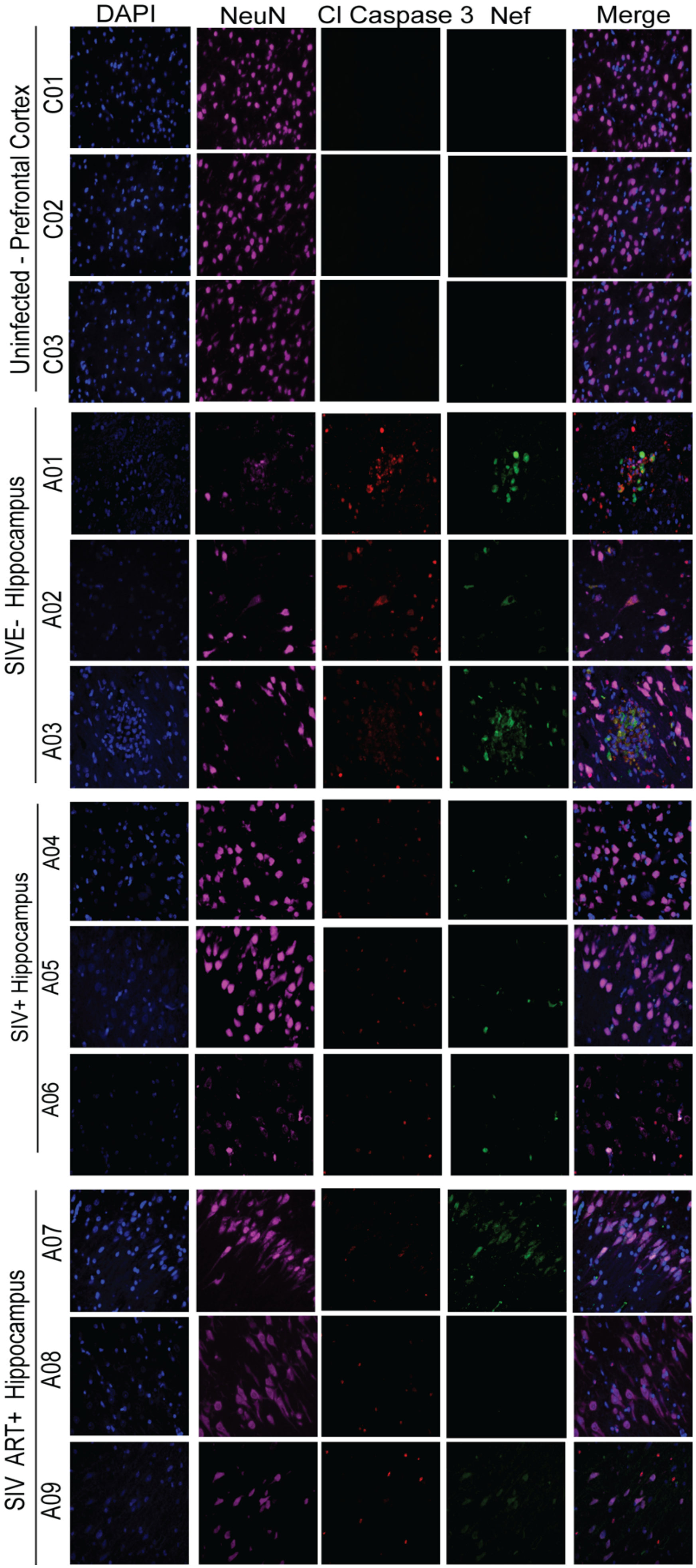

Supplementary figure 8. Multiplex analyses of Nef and cleaved caspase-3 expression in neurons. Pre-frontal cortex (uninfected) and hippocampus regions from paraffin embedded SIV-infected macaques brain samples were serial sectioned and stained for cell specific biomarker NeuN (pink), Cleaved caspase 3 (Red) and Nef (green) using 4 color opal multiplex immunohistochemical assay staining. (Perkin Elmer). Images were taken at 20X magnification with a Keyence BZ-X700 microscope. (n= 3 per group).
